# Smaller anterior hippocampal subfields in the early stage of psychosis

**DOI:** 10.1038/s41398-023-02719-5

**Published:** 2024-01-31

**Authors:** Maureen McHugo, Maxwell J. Roeske, Simon N. Vandekar, Kristan Armstrong, Suzanne N. Avery, Stephan Heckers

**Affiliations:** 1 Department of Psychiatry and Behavioral Sciences, Vanderbilt University Medical Center, Nashville, TN USA; 2 Department of Biostatistics, Vanderbilt University Medical Center, Nashville, TN USA

**Keywords:** Neuroscience, Schizophrenia

## Abstract

Hippocampal volume is smaller in schizophrenia, but it is unclear when in the illness the changes appear and whether specific regions (anterior, posterior) and subfields (CA1, CA2/3, dentate gyrus, subiculum) are affected. Here, we used a high-resolution T2-weighted sequence specialized for imaging hippocampal subfields to test the hypothesis that anterior CA1 volume is lower in early psychosis. We measured subfield volumes across hippocampal regions in a group of 90 individuals in the early stage of a non-affective psychotic disorder and 70 demographically similar healthy individuals. We observed smaller volume in the anterior CA1 and dentate gyrus subfields in the early psychosis group. Our findings support models that implicate anterior CA1 and dentate gyrus subfield deficits in the mechanism of psychosis.

## Introduction

Structural abnormalities of the hippocampus are robust findings in both post-mortem [1] and neuroimaging [2] studies of schizophrenia. Total hippocampal volume is smaller in chronic [3, 4] and early stages [5] of the illness and may decline with illness progression [6, 7]. Smaller volume is already apparent in individuals at high risk for psychosis [8], but it is unclear whether hippocampal volume predicts transition to psychosis [9]. Understanding the specificity and time course of hippocampal volume change is a critical step toward developing better staging models for psychosis.

Current neurobiological models of schizophrenia propose hippocampal dysfunction as a key mechanistic factor of the illness [10, 11], with a focus on pathology in cornu ammonis sectors CA1 [12, 13] and CA2/3 [14] and the dentate gyrus (DG) [15]. Hypermetabolism within these subfields is posited to drive the onset of psychosis and hippocampal volume loss due to excitotoxic spreading [10, 12, 16–18]. Persons with chronic schizophrenia show smaller volume across multiple hippocampal subfields [19–26], a pattern that may also be present in early-stage individuals [16, 27]. However, several studies of at-risk and early-stage individuals show evidence of an *initial* volume deficit in two subfields: the CA1 sector [19, 28–30] and the dentate gyrus [16, 22, 24, 31], reviewed in [32]. The mechanism behind spreading of hippocampal hyperactivity may depend on neurotransmission between CA1 and the dentate gyrus [33]. Consequently, identifying when structural changes occur within these subfields is necessary to inform development of targeted treatments to reduce hyperactivity [34] .

A comprehensive account of hippocampal volume deficits must also consider anterior-posterior gradients of hippocampal function along the long axis [35]. The anterior hippocampus has distinct connectivity with regions involved in processing emotions and motivation and represents more global aspects of the environment compared to the posterior hippocampus [36]. The anterior CA1 region in particular may be more vulnerable to oxidative stress [37], hypoxia [38], and excitability [39] due to differences in gene and NMDA receptor expression patterns [40]. Anterior CA1 hippocampal hypermetabolism is consistently found in psychosis [12, 41–44] and the resulting excitotoxicity may lead to disproportionately lower volume observed in the anterior region [45–49]. Limited evidence suggests that the anterior hippocampus is more affected in the early stages of psychosis [30, 45, 48, 50], but widespread changes across both anterior and posterior regions are present in chronic schizophrenia [21, 25, 51–53] consistent with neuroprogressive pathology [33, 54].

Few studies have examined subfield changes along the hippocampal long axis in early psychosis. While two reports from our group point to selective reduction in the anterior CA subfields [21, 30], a recent study in a large early psychosis cohort suggests that volume deficits may also be present in the posterior subiculum [50] and none of these reports separately measured the CA1 and CA2/3 subfields. High-resolution (sub-millimeter voxels in the coronal plane along the hippocampal long axis), T2-weighted scans acquired at 3 T or 7 T field strength are being used to establish harmonized definitions of subfield boundaries across disciplines [55, 56]. These scans have greater gray/white matter contrast that permits differentiation of the cornu ammonis and dentate gyrus subfields [57] and provide a complementary method to analyses of standard (1mm^3^) resolution images. To our knowledge, only a single study has examined subfield volumes using high in-plane resolution imaging of the hippocampus in a small group of schizophrenia patients, finding smaller CA1 and dentate gyrus volumes [20], but did not consider differences in anterior and posterior regions. High-resolution imaging of individuals in the early stage of psychosis will enable more precise anatomical characterization of the timing of subfield-specific changes, may help to resolve discrepancies between existing studies, and yield greater insight into the impact of aging [58] and antipsychotic medication [59, 60].

Taken together, current evidence suggests that the anterior CA1 subfield is affected in the early stages of schizophrenia. So far, this has only been tested with standard resolution neuroimaging. Here, we use high-resolution structural MRI in a large group of individuals in the early stage of psychosis to test the hypothesis of smaller anterior CA1 volume in the early stage of psychosis. Incomplete hippocampal inversion, a marker of atypical neurodevelopment, has been shown to impact hippocampal volume in large-scale studies of healthy individuals [61] and in schizophrenia [62, 63]. Consequently, we carried out a secondary analysis to test the effect of incomplete hippocampal inversion on hippocampal subfield volumes. Finally, we conducted exploratory analyses to examine whether subfield volume deficits observed in early psychosis were associated with psychosis symptoms, illness duration, or memory deficits.

## Methods

### Participants

Participants were 90 individuals in the early stage of a non-affective psychotic disorder (EP) and 70 demographically similar healthy control individuals (HC), recruited between May 2013 and January 2020 for a prospective longitudinal study on hippocampal structure and function in the early stages of psychosis. EP individuals were recruited from the inpatient and outpatient clinics at Vanderbilt University Medical Center Psychiatric Hospital. HC individuals were recruited from the surrounding community through advertisements. Inclusion criteria were (1) age 13–40; (2) estimated premorbid IQ greater than or equal to 75; (3) <2 years of psychotic illness; and 4) meeting criterion A for schizophrenia (at least two of the following symptoms for a minimum duration of one month: delusions, hallucinations, disorganized speech, disorganized or catatonic behavior, and negative symptoms). Exclusionary criteria for all participants included the presence of significant head injury, major medical illnesses, or pregnancy. EP participants were excluded for active substance abuse or dependence in the past month or diagnosis of a psychotic disorder due to a medical condition. HC participants were excluded if they met criteria for any Axis I disorder or had a first-degree relative with a known psychotic disorder. Data from a subset of participants in this cohort have been included in previous reports on hippocampal volume (N overlap = 122) [21, 30] and incomplete hippocampal inversion (N overlap = 131) [62, 64], but the T2-weighted scans and analyses presented here are novel. All participants provided written informed consent and received monetary compensation for their time. The Vanderbilt University Institutional Review Board approved the study.

### Clinical and cognitive characterization

Demographic and clinical characteristics of participants included in statistical analyses are summarized in Table 1. Psychiatric diagnoses were assessed with the Structured Clinical Interview for DSM-IV, TR (SCID [65]). Data obtained from in-person interviews were augmented by extensive review of all available medical records. Taking into account all available information, diagnostic consensus meetings were held, and final diagnoses were made by psychiatrist SH. The Positive and Negative Symptom Scale (PANSS [66]) was used to characterize clinical symptoms at the time of the scan. Premorbid IQ was estimated using the Wechsler Test of Adult Reading (WTAR [67]). The onset of psychosis was determined through the SCID and Symptom Onset in Schizophrenia Inventory, a standardized measure for rating prodromal versus psychotic symptoms [68]. The duration of psychosis was calculated as the amount of time between the date of onset of psychosis and the scan. Chlorpromazine equivalents were calculated using the formulas from Gardner et al. [69] and Leucht et al. [70] (mean = 329.01, sd = 176.38).

**Table 1:** Demographics and clinical characteristics.

	Healthy control		Early psychosis		Healthy control > Early psychosis		
	*N* = 67		*N* = 86				
	Mean	SD	Mean	SD	t Statistic	df	*p*
Age (years)	21.91	2.96	21.49	3.86	0.77	151	0.44
Parental education (years)	15.12	2.39	14.97	2.76	0.35	149	0.73
WTAR^a^	112.72	10.39	103.81	15.35	4.15	141	<0.001
PANSS							
Positive			17.13	7.23			
Negative			16.21	7.67			
General			32.30	9.59			
Duration of psychosis (months)			7.59	6.39			
CPZ equivalents^b^			329.01	176.38			

	*N*		*N*		*Χ*^2^ Statistic	df	*p*
Gender (Male/Female)	50/17		64/22		0.00	1	0.98
Race (White/Black/Other)	52/11/4		59/25/2		4.26	2	0.12
Number medicated with APD			71				
Diagnosis							
Brief psychotic disorder			2				
Schizophreniform disorder			35				
Schizoaffective disorder			8				
Schizophrenia			41				

*WTAR* Wechsler Test of Adult Reading, *PANSS* Positive and Negative Symptom Scale, *CPZ* chlorpromazine, *APD* antipsychotic drug.

^a^WTAR unavailable for six HC and three EP participants.

^b^Medication information unavailable for one EP participant.

### Relational memory measures

We examined the relationship of volume in the EP group with two measures of relational memory, a hippocampal-dependent cognitive function that is impaired in schizophrenia [71]. A subset of participants completed two tasks to measure relational memory: the face-scene binding task, which uses implicit eye movements to measure relational binding ability, and the associative inference task, which measures the ability to make explicit inferences about previously bound pairs of items (detailed descriptions of each task and how behavioral measures were calculated are presented in [72]). From the face-scene binding task, we calculated viewing slope (*n* = 74; smaller slope = worse performance; mean slope=11.63, sd = 12.14). For associative inference, we examined inference pair accuracy in participants who achieved at least 80% accuracy during training (*n* = 71 included; mean accuracy = 0.74, sd = 0.19; data from an additional 14 EP participants were excluded for failing to pass training criteria).

### Data acquisition and processing

Structural imaging data were collected on one of two identical 3 T Philips Intera Achieva scanners with a 32-channel head coil (Philips Healthcare, Inc., Best, The Netherlands) at the Vanderbilt University Institute of Imaging Sciences. We acquired a 3D T1-weighted image (voxel size = 1mm^3^; field of view = 256 mm^2^; number of slices = 170; gap = 0 mm; TE = 3.7 ms; TR = 8.0 ms) and a T2-weighted turbo spin echo scan with oblique coronal slices oriented perpendicular to the hippocampal long axis (in-plane resolution = 0.5mm^2^; slice thickness = 2 mm; field of view = 230 mm x 184 mm; number of slices =26; gap = 0 mm; TE = 90 ms; TR = 2375 ms).

**Fig. 1:**
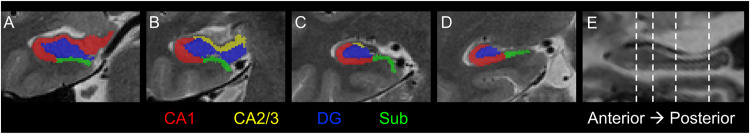
Segmentation of subfields in the anterior and posterior hippocampus. Coronal slices taken through the hippocampus from anterior (**A**, **B**) to posterior (**C**, **D**) with subfields labeled by color. **E** Sagittal slice showing the longitudinal axis of the hippocampus with dashed lines indicating the position of each coronal slice from (**A**–**D**).

Hippocampal subfield volumes were obtained from the T2 and T1-weighted images using the Automated Segmentation of Hippocampal Subfields (ASHS) software with the Penn PMC atlas [73]. ASHS uses a library of manually segmented atlas images to automatically label hippocampal subfields in a participant’s native space (Fig. 1). Each participant’s T2 and T1 images are rigidly aligned and then registered to a T1 template image. All T2 atlas images are then registered to the participant’s T2 image. Multi-atlas joint label fusion is used to produce an initial consensus segmentation of the participant T2 image and corrective learning is used as a post-processing step to adjust segmentation errors. The resulting hippocampal segmentations were manually divided into anterior and posterior regions based on the presence of an anatomical landmark, the uncus [36, 74, 75]. The final slice of the anterior region was defined as the last coronal slice in which there were two cuts visible through the hippocampus, such that the uncus itself would have been included in the anterior region. Quality control consisted of visual inspection by author MM. T1 and T2-weighted images were visually inspected for motion artifacts and hippocampal segmentations were inspected for mislabeling. Segmentations that had voxels labeled outside the hippocampus (in the amygdala, adjacent white matter, or ventricles) or in which the hippocampus was not completely labeled were reviewed with expert rater SH before being excluded (N excluded = 4 EP and 3 HC individuals). No manual editing of segmentations was carried out. We obtained estimated total intracranial volume using Freesurfer 6 [76, 77].

The presence and severity of incomplete hippocampal inversion were determined using previously published criteria [61] by a single rater (MR). Each hippocampus was scored for incomplete inversion (range 0–10, higher score = more severe incomplete hippocampal inversion) and categorized as having incomplete hippocampal inversion (IHI) based on a cutoff score of ≥3.75 (described in [62]). We identified 29 cases of unilateral left hemisphere IHI, 2 cases of unilateral right hemisphere IHI, and 8 cases of bilateral IHI in our sample; 114 individuals did not have IHI (Table 2).

**Table 2:** Incomplete hippocampal inversion (IHI) frequency and severity.

	Healthy control		Early psychosis		Healthy control > Early psychosis		
	*N* = 67		*N* = 86				
	Mean	SD	Mean	SD	*t* Statistic	df	*p*
IHI score							
Left	2.51	1.07	3.03	1.68	−2.32	146	0.02
Right	1.90	0.79	2.15	1.10	−1.63	150	0.11

	*N*	%	*N*	%	*Χ*^2^ Statistic	df	*p*
IHI present (yes/no)							
Left	10/57	15/85	27/59	31/69	5.57	1	0.02
Right	2/65	3/97	8/78	9/91	2.46	1	0.12

### Statistical analysis

Statistical analysis of hippocampal subfield volume was carried out using linear mixed models in R (R Core Team, 2019) with the packages *lmerTest* [78], *emmeans* [79], and *car* [80]. To test whether there are regionally specific subfield volume deficits in early psychosis, we fitted a model with Volume as the outcome variable and Group (healthy control, early psychosis), Hemisphere (left, right), Region (anterior, posterior), Subfield (CA1, DG, subiculum, CA2/3), and their interaction as fixed effects, and participant as a random effect (Model 1: Volume ~ Group x Hemisphere x Region x Subfield + Age + Sex + ICV + Scanner + (1|Participant)). Age, sex, intracranial volume, and scanner were included as covariates of no interest. We conducted significance tests on the fixed effects in each model using analysis of variance (ANOVA) on the model output. Significant effects were followed up with contrasts adjusted for multiple comparisons using Bonferroni correction. Model assumptions were examined using fitted vs. residual plots, scale location plots, quantile-quantile plots, and the variance inflation factor (all values < 2). The assumptions of normality of residuals and homogeneity of variance were violated in the full model. Although linear mixed models are robust to violations of these assumptions [81], we fitted separate linear mixed models for each subfield (four models: CA1, CA2/3, DG, Subiculum) that examined the effects of Group, Hemisphere, and Region and are detailed in the supplement. After correction for four multiple comparisons, the results did not differ from those of the full model. A secondary model including incomplete hippocampal inversion score was fitted to examine whether it contributes to hippocampal subfield volume differences in early psychosis (Model 2: Volume ~ Group × Hemisphere × Region x Subfield + IHI + Age + Sex + ICV + Scanner + (1|Participant)).

Exploratory analyses examining the relationship between volume with clinical and cognitive characteristics in the EP group were carried out using linear regression. We used separate linear regression models for anterior CA1 and DG to test for an association between volume and positive, negative, and general PANSS scores, duration of psychosis, chlorpromazine equivalents, FSB viewing slope, and AI accuracy. All analyses included intracranial volume and scanner as covariates of no interest.

## Results

### Hippocampal subfield volume analysis

**Fig. 2:**
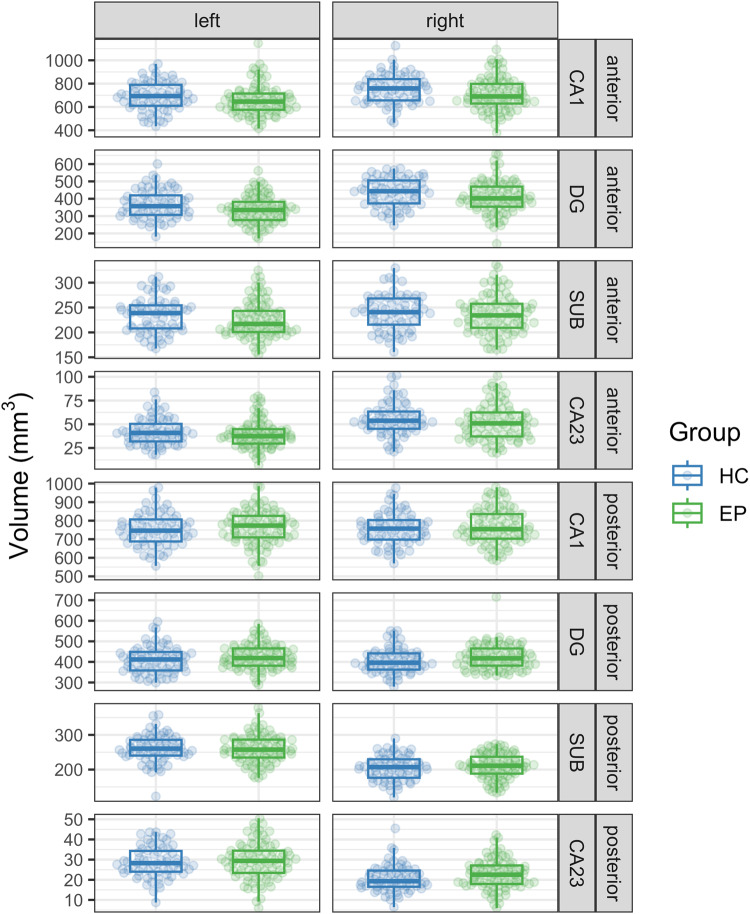
Lower volume in anterior CA1 and anterior DG subfields in early psychosis compared to healthy controls.

We found group differences in subfield volumes in the anterior region of the hippocampus (Fig. 2; Group × Region × Subfield interaction: *F*_3,2261_ = 5.66, *p* < 0.001). Individuals in the early stage of psychosis showed lower volume than healthy controls in the anterior CA1 (*t*_960_ = −5.06, *p* < 0.001) and DG (*t*_960_ = −3.96, *p* = 0.001) subfields, but not in CA2/3 (*t*_968_ = −0.80, *p* = 1.00) or the subiculum (*t*_960_ = −1.20, *p* = 1.00). We did not find evidence for group differences in volume for any subfields in the posterior hippocampus (all *p*’s > 0.92). In a secondary analysis with incomplete hippocampal inversion included in the model, we found a main effect of incomplete hippocampal inversion (*F*_1,1048_ = 8.69, *p* = 0.003), but our primary results remained unchanged (Group × Region × Subfield interaction: *F*_3,2260_ = 5.67, *p* < 0.001).

### Associations with clinical characteristics and memory performance

We did not find evidence that anterior CA1 and DG volumes were associated with psychosis symptoms, duration of psychosis, chlorpromazine equivalents, or relational memory performance in the EP group (Table 3).

**Table 3:** Association of clinical characteristics and memory performance with mean anterior CA1 and DG volumes.

	Anterior CA1 volume		Anterior DG volume	
	t Statistic	*p*	t Statistic	*p*
PANSS				
Positive	0.70	0.49	1.27	0.21
Negative	1.02	0.31	0.35	0.73
General	−0.17	0.87	−0.48	0.63
Psychosis duration	0.69	0.50	0.75	0.45
Chlorpromazine equivalents	0.27	0.79	−0.38	0.71
Face-Scene Binding (viewing slope)^a^	−0.58	0.56	0.18	0.86
Associative Inference (accuracy)^b^	−0.28	0.78	−0.52	0.60

^a^Face-scene binding task data included from 74 EP participants.

^b^Associative inference task data included from 71 EP participants.

## Discussion

In a large cross-sectional study, we show that hippocampal volume deficits are limited to the CA1 and DG subfields in the anterior region in the early stage of psychosis. To our knowledge, this is only the second study in schizophrenia to use high-resolution structural imaging designed to maximize visualization of anatomical detail within the hippocampus [20] and the first of its kind in an early psychosis cohort. First, we will discuss the value of high-resolution imaging in the study of hippocampal volume in schizophrenia. Then we will review functional implications of regionally specific hippocampal volume changes in the early stage of psychosis.

A post-mortem study of schizophrenia was the first to report smaller hippocampal volume [82]. The initial wave of CT and MRI studies confirmed smaller *total* hippocampal volume in schizophrenia [5], which is now recognized as the largest effect size among the numerous structural brain abnormalities observed in patients [3]. In a second wave, MRI studies explored hippocampal *subfield* volumes in schizophrenia [26]. But in contrast to post-mortem studies, which employed cytoarchitectural criteria to study hippocampal subfields, such detail is not available for neuroimaging studies. Therefore, researchers employed protocols for automated segmentation of subfields in the human hippocampus. The initial versions of these protocols arrived at volume estimates that are not compatible with the known anatomy of the human hippocampus [83]. Subfield volumes derived from 1mm^3^ resolution images is further limited by an inability to visualize internal details of hippocampal structure necessary for differentiation of the cornu ammonis and dentate gyrus subfields and automated methods applied to these data may primarily reflect differences in overall volume [57]. Consequently, some reports of subfield-specific deficits of hippocampal volume in schizophrenia from this second wave need to be interpreted with caution.

Our study belongs to the third wave of neuroimaging studies exploring hippocampal volume differences in schizophrenia using advanced imaging and segmentation methods. Accurate inferences regarding the nature of subfield-specific volume deficits in schizophrenia require valid, reliable, and reproducible methods that can be applied to large-scale datasets. T2-weighted coronal images of the hippocampus with high in-plane resolution (0.4–0.5 mm) enable delineation of the cornu ammonis and dentate gyrus and are recommended by consensus groups dedicated to harmonizing subfield segmentation protocols across laboratories [55]. While manual segmentation of subfields on such images remains the current best practice, the use of automated methods that are validated against manual segmentations will facilitate research on subfield structure in large datasets [57]. We included a high-resolution protocol using T1 and T2-weighted images, employed an accepted anatomical criterion to define anterior–posterior regions, and included the classification of incomplete hippocampal inversion, a variant in the development of the human hippocampus. Future meta-analytic studies of hippocampal volume in schizophrenia will need to consider the confounding effects of data acquisition and hippocampal segmentation.

In contrast to most previous studies of hippocampal volume in schizophrenia, we specifically tested for volume differences in the anterior region. Recent work has highlighted distinct functions of both hippocampal subfields (transverse axis) and hippocampal regions (longitudinal axis) [36, 84]. In fact, a full account of hippocampal function may be described best by gradients across both axes [35, 40]. Our data indicate smaller subfield volumes in the anterior but not posterior region in schizophrenia. Recent data-driven parcellations of the hippocampus based on both functional activation and gene expression patterns have provided convergent evidence that the anterior hippocampus is primarily involved in processing affective, motivational, and self-relevant information [85, 86]. Dysregulation of mood and affect are among the earliest symptoms to emerge during the prodromal period [87] and disturbance of self-related cognition may be a core feature of schizophrenia [88]. While we have taken an approach of using anatomically defined markers of hippocampal subregions in the current work, future studies are needed that jointly consider individual-specific parcellations of structural and functional data and their relationship to psychosis symptoms and cognition.

We confirm a prior study that highlighted the importance of CA1 and DG subfield abnormalities in the early stage of psychosis [32]. Our finding of smaller CA1 and DG volumes is of significant functional importance and lends support to several existing models of hippocampal dysfunction in schizophrenia. CA1 hyperactivity arising from glutamatergic dysfunction [12] or deficits in GABAergic interneurons [89] is thought to lead to positive symptoms and cognitive impairments. Early hyperactivity within CA1 may then spread in an excitotoxic cascade to adjacent subfields with illness progression, ultimately leading to concomitant volume deficits [17, 54]. A separate line of research suggests that reduced glutamatergic signaling in the DG gives rise to memory deficits and positive symptoms [15]. However, we did not find evidence for CA2/3 subfield changes [14, 90] in our cohort. Future longitudinal studies of hippocampal function, in concert with the type of structural neuroimaging presented here, are needed to fully determine a causal link between hippocampal hyperactivity and atrophy [29].

While incomplete hippocampal inversion explained substantial variance in hippocampal subfield volumes, we did not find evidence that it differentially impacted our primary finding of volume deficits within the anterior CA1 and DG subfields of EP individuals. Although we had a large cohort of individuals in the early stage of psychosis in the present study, the relatively low incidence of incomplete hippocampal inversion may have precluded our ability to observe a differential effect by group. Hemispheric asymmetry in the hippocampus is well-described [74] and incomplete hippocampal inversion contributes to asymmetry across healthy individuals and those with psychosis [62]. Unfortunately, because of the low incidence of right incomplete hippocampal inversion (2 HC, 8 EP), the current sample is underpowered to fully examine the question of hemispheric differences in subfield volumes across groups and anterior–posterior regions. Analysis of incomplete hippocampal inversion in larger or consortia datasets is needed to examine its relationship to hippocampal subfield volumes across the anterior-posterior axis.

We did not find evidence of an association between anterior CA1 or DG subfield volumes and clinical characteristics or memory performance in this sample. As we have discussed previously [30], our sample was identified very early in the illness (mean duration of psychosis ~8 months). It is possible that there is too little variation within the variables examined to observe a relationship with the subtle volume deficits that are present in the early stage of illness. The relationship between hippocampal dysfunction, psychosis symptoms, and memory impairment is likely complex and multifactorial. Future studies are needed that examine information about hippocampal structure and function (e.g., connectivity, perfusion, task-based fMRI) together with clinical and cognitive measures.

While high-resolution imaging in a large cohort is a strength of our study, there are also limitations. First, the cross-sectional data presented here are from a cohort of individuals in the early stage of illness. Not all non-affective psychotic disorder patients included in our cohort will progress to schizophrenia [91]. Additionally, hippocampal volume continues to change throughout the lifespan, particularly within the cornu ammonis and dentate gyrus subfields [92] and may exhibit nonlinear changes that differ in anterior and posterior regions in the age range represented in this sample [93, 94]. Longitudinal imaging is needed to clarify how hippocampal subregion volumes vary with clinical and diagnostic trajectory and the extent to which the differences in hippocampal subfield volumes from the present study reflect early neurodevelopmental processes or ongoing illness progression in psychosis. Second, while we did not find evidence for volume changes in the CA2/3 or subiculum, segmentation of these small subfields is challenging, even with manual segmentation [73]. Ultra-high-resolution 7 T imaging is needed to confirm the present findings. Finally, the majority of patients in our study were on antipsychotic medication and we cannot rule out their impact on hippocampal volume [95]. Although antipsychotic treatment may have greater effects within the dentate gyrus [60], data from antipsychotic-naïve individuals suggests that lower volume is not due solely to medication [16].

In summary, we find compelling evidence for subfield-specific changes in the anterior, but not posterior, hippocampus in the early stage of non-affective psychosis. These findings indicate that the more pervasive changes of hippocampal structure present in chronic schizophrenia are not yet established in the first two years of illness. Novel interventions and treatments aimed at normalizing hippocampal function may offer a pathway to preserving hippocampal volume and improving functional outcomes in non-affective psychosis [10].

### Supplementary information

Supplemental material

## Data Availability

The data used in the current study are available by reasonable request to the corresponding author.
