# Supplementary material for: Smaller anterior hippocampal subfields in the early stage of psychosis
Source: Transl Psychiatry. 2024 Jan 31;14:69. doi: 10.1038/s41398-023-02719-5 (PMC10830481; doi:10.1038/s41398-023-02719-5)
Supplement: Supplementary file 1 — Supplemental material [file 41398_2023_2719_MOESM1_ESM.docx]

**Statistical Analysis**

In our previous work, we observed a moderate-to-large effect size for volume differences between individuals with psychosis and healthy controls (Cohen’s d=0.74). Using a rejection threshold of alpha=0.05, we expect to be able to detect this effect with at least 80% power for group sizes of 49 early psychosis and 49 healthy controls (actual sample sizes: 86 early psychosis and 67 healthy controls). In our primary analysis to test whether there are regionally specific subfield volume deficits in early psychosis, we fitted a model with Volume as the outcome variable and Group (healthy control, early psychosis), Hemisphere (left, right), Region (anterior, posterior), Subfield (CA1, DG, subiculum, CA2/3), and their interaction as fixed effects, and participant as a random effect (Model 1: Volume ~ Group x Hemisphere x Region x Subfield + Age + Sex + ICV + Scanner + (1|Participant)). The assumptions of normality of residuals and homogeneity of variance were violated in the full model. To confirm that these violations did not affect our results and conclusions, we fitted separate linear mixed models for each subfield (four models: CA1, CA2/3, DG, Subiculum) that examined the effects of Group, Hemisphere, and Region as fixed effects, and participant as a random effect (Subfield Volume ~ Group x Hemisphere x Region + Age + Sex + ICV + Scanner + (1|Participant)). Age, sex, intracranial volume, and scanner were included as covariates of no interest. No assumptions were violated for the limited models that separately examined CA1, DG, and the subiculum. However, the residuals for the model examining CA2/3 were not normally distributed and the values were then square root transformed. After adjusting for four tests, we observed a significant Group X Region interaction in CA1 (p=0.003) and DG (p<0.001), but not CA2/3 (p=0.08) or the subiculum (p=0.24).
